# USP38 exacerbates pressure overload-induced left ventricular electrical remodeling

**DOI:** 10.1186/s10020-024-00846-3

**Published:** 2024-06-27

**Authors:** Yucheng Pan, Zheng Xiao, Hongjie Yang, Bin Kong, Hong Meng, Wei Shuai, He Huang

**Affiliations:** 1 Department of Cardiology, Renmin Hospital of Wuhan University, 238 Jiefang Road, Wuhan, 430060 Hubei China; 2 Hubei Key Laboratory of Cardiology, Wuhan, China; 3 Cardiovascular Research Institute of Wuhan University, Wuhan, China

**Keywords:** Ubiquitin-specific protease 38, Ventricular arrhythmias, Heart failure, Electrical remodeling

## Abstract

**Background:**

Ubiquitin-specific protease 38 (USP38), belonging to the USP family, is recognized for its role in controlling protein degradation and diverse biological processes. Ventricular arrhythmias (VAs) following heart failure (HF) are closely linked to ventricular electrical remodeling, yet the specific mechanisms underlying VAs in HF remain inadequately explored. In this study, we examined the impact of USP38 on VAs in pressure overload-induced HF.

**Methods:**

Cardiac-specific USP38 knockout mice, cardiac-specific USP38 transgenic mice and their matched control littermates developed HF induced by aortic banding (AB) surgery. After subjecting the mice to AB surgery for a duration of four weeks, comprehensive investigations were conducted, including pathological analysis and electrophysiological assessments, along with molecular analyses.

**Results:**

We observed increased USP38 expression in the left ventricle of mice with HF. Electrocardiogram showed that the USP38 knockout shortened the QRS interval and QTc, while USP38 overexpression prolonged these parameters. USP38 knockout decreased the susceptibility of VAs by shortening action potential duration (APD) and prolonging effective refractory period (ERP). In addition, USP38 knockout increased ion channel and Cx43 expression in ventricle. On the contrary, the increased susceptibility of VAs and the decreased expression of ventricular ion channels and Cx43 were observed with USP38 overexpression. In both in vivo and in vitro experiments, USP38 knockout inhibited TBK1/AKT/CAMKII signaling, whereas USP38 overexpression activated this pathway.

**Conclusion:**

Our data indicates that USP38 increases susceptibility to VAs after HF through TBK1/AKT/CAMKII signaling pathway, Consequently, USP38 may emerge as a promising therapeutic target for managing VAs following HF.

**Supplementary Information:**

The online version contains supplementary material available at 10.1186/s10020-024-00846-3.

## Introduction

Heart failure (HF) is the ultimate destination of cardiovascular diseases, impacting the health of millions worldwide and placing a substantial burden on healthcare systems (Tsao et al.^2022^). Sustained ventricular pressure overload stimuli, such as hypertension, vascular disease, and aortic stenosis, can lead to pathological myocardial remodeling and dysfunction, serving as a precursor to clinical heart failure (Kehat and Molkentin 2010; Wu et al. 2017). Structural and functional changes in HF are considered substrates for ventricular arrhythmias (VAs) (Zhang et al.^2018^). Studies also have reported that VAs occur in up to 30%-50% of HF cases, mainly including ventricular tachycardia (VT) and ventricular fibrillation (VF) (Santangeli et al.^2017^). Electrical remodeling, characterized by prolonged action potential duration (APD) and ion channel abnormalities, forms the basis for the pathogenesis of VAs (Varró et al.^2021^). However, the underlying mechanisms leading to the development of VAs following HF remains incomplete.

Ubiquitin specific proteases (USPs), potential regulators of intracellular protein levels and functions, are known to regulate protein degradation and various biological processes. USPs can removes ubiquitin chains from ubiquitin tagged proteins, thereby stabilizing intracellular proteins, and influencing cellular signaling pathways (Lill and Wertz 2014). While the role of USPs have been extensively studied in different diseases, their implications in cardiovascular conditions, especially arrhythmias, remain poorly understood. USP38, a member of the USP family, has been associated with respiratory diseases and the tumor development (Yi et al. 2022; Yang et al. 2020b, a). Previous studies found that USP38 can interact with the TBK1 through a ubiquitin-protease pathway (Lin et al. 2016). TBK1 initiates the activation of AKT signaling in mice undergoing aortic ligation surgery, and this pathway is implicated in the progression of cardiac remodeling (Deng et al. 2016). Interestingly, the occurrence of arrhythmias is intricately connected to the interplay between AKT and CAMKII (Mustroph et al. 2017; Pereira et al. 2017). Given the limited knowledge of USP38 in cardiovascular diseases, our study aims to investigate its role in cardiac electrical remodeling and its potential involvement in VAs following HF.

In this study, we generated cardiac-specific USP38 transgenic mice (USP38-TG) and cardiac-specific USP38 knockout mice (USP38-CKO) to investigate the role and molecular mechanism of USP38 in VAs during HF induced by pressure overload. The results revealed that USP38-TG mice subjected to aortic banding (AB) surgery exhibited increased vulnerability to VAs and displayed maladjustment in the expression of Cx43 and ion channels proteins. Conversely, USP38-CKO mice subjected to AB surgery displayed the opposite phenotype. The molecular mechanism of VAs might be attributed to the action of USP38 on TBK1, AKT and CAMKII signaling molecules. This research might provide a basis for novel therapeutic strategies to reduce the incidence of VAs caused by HF.

## Materials and methods

### Animals and animal model

All animal care and experimental procedures were performed in accordance with the National Institutes of Health Guide for the Care and Use of Laboratory Animals. In addition, this study received approval from Animal Care and Use Committee of Renmin Hospital of Wuhan University (20221207B). The cardiac-specific USP38 knockout and transgenic mice (C57BL/6 background) were obtained from the Cyagen Biotechnology using the Cre-loxp system technology. mice were housed in a specific pathogen-free (SPF) environment with a temperature under a 12-h light/dark cycle, with food and water available.

The AB surgery was performed as previously described (Wu et al. 2018). Briefly, mice were anesthetized with 3% pentobarbital sodium at a dose of 40 mg/kg. The thorax of the mouse was exposed, and the thoracic aorta was found and ligated with a 27G needle using a 7–0 silk suture. A 70% constriction was achieved when the blunt needle was withdrawn after ligation. A similar operation was performed without ligating the aorta for the sham operation group. The wild-type mice were divided into two groups: Sham groups, AB groups (n = 12 in each group). Mice with heart specific knockout of USP38 were divided into four groups: Flox-Sham groups, CKO-Sham groups, Flox-AB groups, CKO-AB groups (n = 20 in each group). Mice with heart specific transgenic of USP38 were divided into four groups: NTG-Sham groups, TG-Sham groups, NTG-AB groups, TG-AB groups (n = 20 in each group).

### Cell culture and treatment

H9c2 cells were cultured in Dulbecco’s Modified Eagle Medium (DMEM, Invitrogen Corporation, USA), supplemented with 10% FBS, streptomycin, penicillin, and incubated in a 5% CO2 atmosphere at 37 °C. Hypertrophy in vitro was induced in H9c2 cells by the addition of 1μM angiotensin II (Ang II). Recombinant adenoviruses were constructed to knock out and overexpress the target gene in H9c2. AdshUSP38 was generated using short hairpin RNAs (shRNAs) for USP38 knockdown, while AdUSP38 was constructed using a replication-defective adenoviral vector containing rat USP38 cDNA. AdshRNA and AdGFP served as controls for AdshUSP38 and AdUSP38 respectively. H9c2 cells were infected with AdshUSP38 and AdshRNA at a multiplicity of infection (MOI) of 40 for 24 h and with AdUSP38 and AdGFP at a MOI of 20 for 24 h. H9c2 cells infected with AdshUSP38 were inoculated on the 6-well plate and divided into four groups: PBS + AdshRNA groups, PBS + AdshUSP38 groups,Ang II + AdshRNA groups, Ang II + AdshUSP38 (n = 6 in each group). H9c2 cells infected with AdUSP38 were inoculated on the 6-well plate and divided into four groups: PBS + AdGFP groups, PBS + AdUSP38 groups, Ang II + AdGFP groups, Ang II + AdUSP38 (n = 6 in each group).

### Echocardiography

After four weeks post-surgery, mice were anesthetized with 1.5% isoflurane, and their cardiac function was assessed using an ultrasound Doppler imaging system (VINNO6, Vinno Corporation, China). The B-Mode was utilized to locate the long-axis view, which was then switched to M-mode to record the myocardial movement over time. Parameters of cardiac function, including the left ventricular ejection fraction (LVEF) and left ventricular fractional shortening (LVFS), were measured (n = 4 in Sham and AB groups, n = 8 in Mice with heart specific knockout and transgenic of USP38 gruops).

### Surface Electrocardiogram (ECG)

Mice were anesthetized with 1.5% isoflurane and placed on a heating plate. Similar to the surface-lead ECG (lead II), mice were implanted with subcutaneous electrodes to record electrocardiogram-related indicators, including RR, PR, QRS, and QT interval. Lab-Chart 8 Pro (AD Instruments) collected the mouse electrocardiogram for 5 min and analyzed the data. To correct for the effects of different heart rates, QTc was calculated using Bazett’s formula: QTc = QT/√RR (n = 8 in each group).

### In vivo electrophysiological study

The mice were anesthetized with 1% pentobarbital sodium, and then the chest was opened to expose the heart. The monophasic action potentials (MAP) of left ventricle were recorded using a platinum MAP electrode and stimulation procedures. A MAP electrode was positioned on the anterior free wall of the left ventricle to record epicardial MAP, accompanied by a paired platinum stimulating electrode on the basal surface of the right ventricle. The electrical stimulation program was performed in a method similar to that previously reported (Yang et al. 2020b, a). The heart was stimulated with a regular pacing cycle length (PCL). Action potential duration (APD) and APD alternans (ALT) were measured by S1-S1 pacing. When the PCL was 150 ms, APD90, APD50 and APD20 were defined as the average repolarization time of 90%, 50% and 20% of 6–8 consecutive MAP under 150 ms PCL (n = 8 in each group). ALT was measured by graded stimulation. PCL was decreased by 10 ms each step from 150 to 100 ms and then by 5 ms from 100 to 50 ms. When the difference between two adjacent MAP is at least 5%, it is considered that the ALT threshold. The ALT threshold is the longest S1-S1 pacing perimeter that induces APD90 alternation (n = 8 in each group). The S1-S2 (8- consecutive stimuli (S1,150 ms) followed by an extra stimulus (S2)) stimulation program was used to determine the effective refractory period (ERP) of the ventricle. When the pairing interval of S1S2 gradually shortens, S2 finally enters the refractory period until ventricular ERP (n = 8 in each group). Burst pacing (5 V, 50 Hz, 2 ms pulse, 2-s burst duration) was used to induce VAs. continuous VT or VF lasting > 2 s was recorded as VAs. The following are the number of groups for conducting burst pacing: Sham groups (n = 10), AB groups (n = 12), Flox-Sham groups (n = 16), CKO-Sham groups (n = 16), Flox-AB groups (n = 18), CKO-AB groups (n = 19), NTG-Sham groups (n = 17), TG-Sham groups (n = 16), NTG-AB groups (n = 19), TG-AB groups (n = 20).

### Wheat germ agglutinin staining

The left ventricle tissue was fixed in 4% paraformaldehyde solution for 24 h. Subsequently, the tissue was dehydrated, embedded in paraffin, and cut into 3–5 μm slices. the cross-sectional area of the myocytes was observed through FITC-conjugated wheat germ agglutinin (WGA) staining (n = 4 in each group).

### Immunofluorescence

Cx43 was assessed by immunofluorescence in ventricular tissue. Frozen heart sections were fixed and incubated with Cx43 (AF0137, affinity), and then incubated with the second antibody. Nucleus were stained with DAPI Staining Solution (Servicebio, China). Fluorescence images are collected using a fluorescent microscope (Nikon Eclipse C1, Japan) (n = 4 in each group).

### Quantitative real-time PCR

RNA from left ventricle and H9c2 cells was extracted using reagent (Invitrogen), and the RNA was transcribed into cDNA with the PrimeScript RT reagent Kit (TaKaRa). qRT-PCR was performed On an Applied Biosystems VII7 instrument (Life Technologies, Carlsbad, USA) to determine USP38 mRNA levels. The primer sequences of USP38 and GAPDH are found in the Supplementary Table S1 (n = 3 in each group).

### Western blotting

Cardiac left ventricular and cellular proteins were extracted on ice by adding phosphatase inhibitors to RIPA lysis buffer (Servicebio, China). Proteins were separated by electrophoresis in 10% SDS-PAGE gels and transferred to a polyvinylidene difluoride (PVDF) membrane (Millipore, America). The membrane was blocked with 5% skimmed milk. Subsequently, the membrane was exposed to the primary antibody and incubated overnight, and the secondary antibodies conjugated with HRP was added and incubated on the shaker for 1 h (n = 3 in each group).

Primary antibody against USP38 (17,767–1-AP) were purchased form proteintech. Primary antibody against phospho-CAMKII (GTX52342) was purchased from Gene Tex. Primary antibody against Kv4.2 (ab123543) and CAMKII (ab134041), were purchased form Abcam. Primary antibodies against Kv1.5 (PA5-77573), Kv2.1 (PA5-77580) and Kv4.3 (PA5-77583) were purchased from Invitrogen. Primary antibody against TBK1 (3504), p-TBK1 (5483), AKT (9272), p-AKT (4060) and the secondary antibody (7074) were purchased form Cell Signaling Technology. Primary antibody against GAPDH was purchased from Servicebio. Primary antibody against Cx43 (AF0137) was purchased from affinity.

### Co-immunoprecipitation

H9c2 cells were collected and treated with immunoprecipitation (IP) lysis buffer, supplemented with PMSF and a cocktail. Following centrifugation of the cell lysate, the supernatant was collected. Anti-USP38, anti-TBK1, or anti-IgG antibodies were added to form immune complexes overnight. The resulting immune complexes were then incubated with magnetic beads at room temperature for 1 h. Subsequently, the magnetic beads were washed with loading buffer, denatured, and eluted for Western blotting experiments, the experiment was repeated three times.

### Statistical analysis

Data were analyzed using GraphPad Prism software and presented as mean ± standard error (SEM) or percentage. To assess the normal distribution of the two data sets, we conducted the Shapiro–Wilk test. An unpaired t-test was used when the data sets adhered to a normal distribution. When the two data sets did not conform to a normal distribution, the data were analyzed using Mann–Whitney test. For multiple data sets conforming to a normal distribution, one-way ANOVA with Tukey’s post hoc analysis was used. Categorical data passed the Fisher's exact test. A level of P < 0.05 was considered statistically significant.

## Results

### USP38 expression is up-regulated in the left ventricle of HF mice and the AngII-stimulated H9c2 cells.

**Fig. 1:**
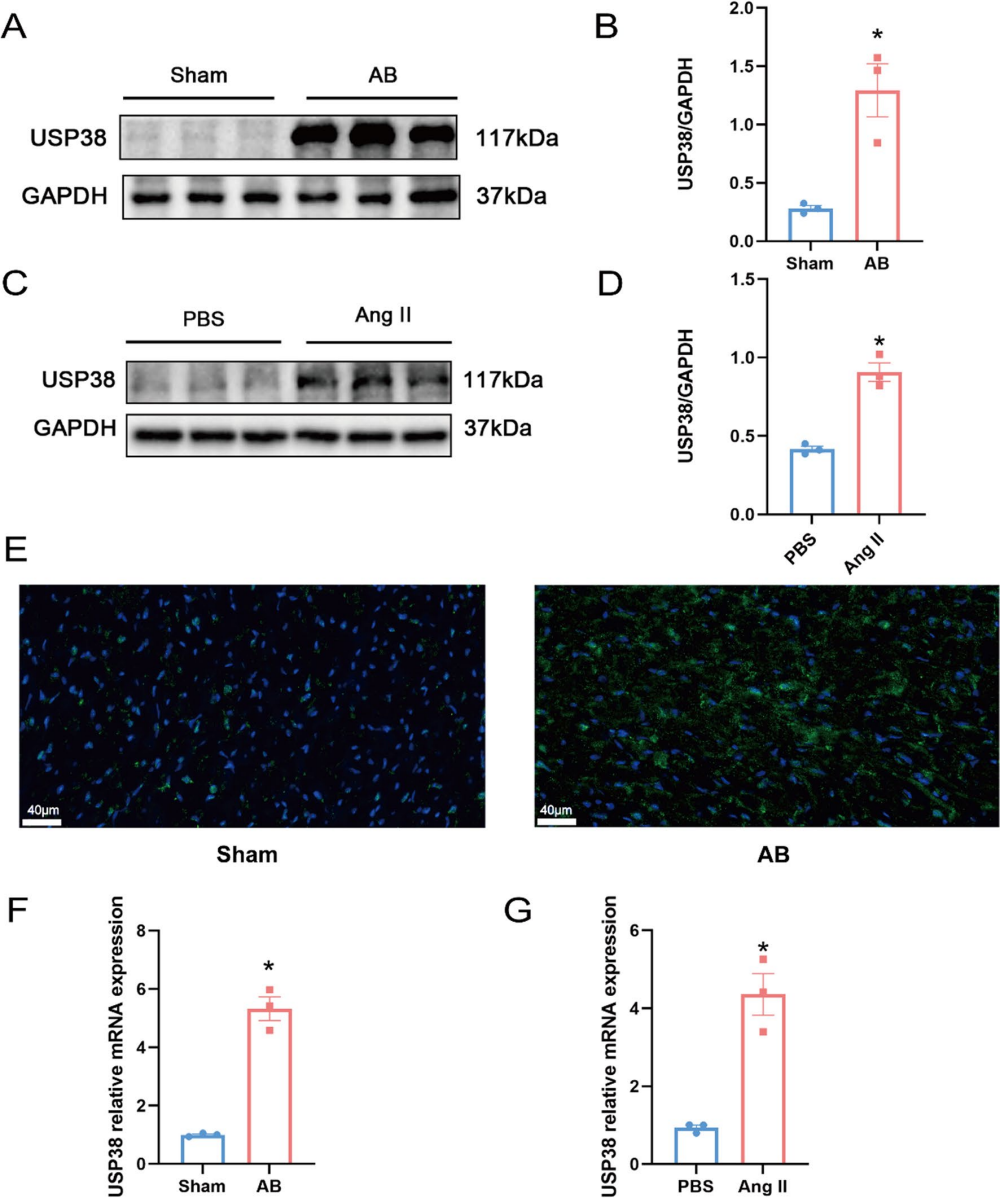
The expression of USP38 is increased with HF. **A**, **B** Mice were exposed to AB surgery for 4 weeks, and then fresh left ventricle were prepared to detect USP38 expression using western blot (n = 3). **C**, **D** Representative western blotting and statistical analysis of USP38 in H9c2 Cell induced by AngII (n = 3). **E** Representative USP38 immunofluorescence staining images in Sham and AB mice (n = 3). **F** Quantitative analysis of USP38 changes in mice 4 weeks after AB by real-time quantitative polymerase chain reaction (n = 3). **G** Quantitative analysis of USP38 changes in H9c2 Cell induced by AngII by real-time quantitative polymerase chain reaction (n = 3). *P < 0.05 vs. Sham group

To explore the correlation between USP38 and HF, we first established HF mice model through AB surgery. Echocardiographic examination showed that the mice after AB surgery had decreased LVEF and LVFS than the WT mice (Fig. S1A, B). Moreover. WAG-stained heart sections confirmed myocardial hypertrophy in AB mice (Fig. S1C, D). We assessed USP38 levels in LV tissues and detected a higher USP38 expression in AB mice (Fig. 1A, B, E). Meanwhile, H9c2 cells were exposed to AngII for 48 h to mimic the hypertrophy in vitro. Similarly, the USP38 expression levels increased in Ang II group compared with the PBS group (Fig. 1C, D). qRT-PCR results indicated a significant upregulation of relative mRNA expression of USP38 in the AB group compared with the Sham group (Fig. 1F). Consistent with the in vivo experimental findings, the relative mRNA levels of USP38 increased in H9c2 induced by AngII (Fig. 1G). Moreover, the susceptibility to VAs increased 4 weeks after AB surgery (Fig. S2A–H).

### USP38 deficiency improves cardiac function and reduces the susceptibility of VAs in mice with HF induced by pressure overload.

**Fig. 2:**
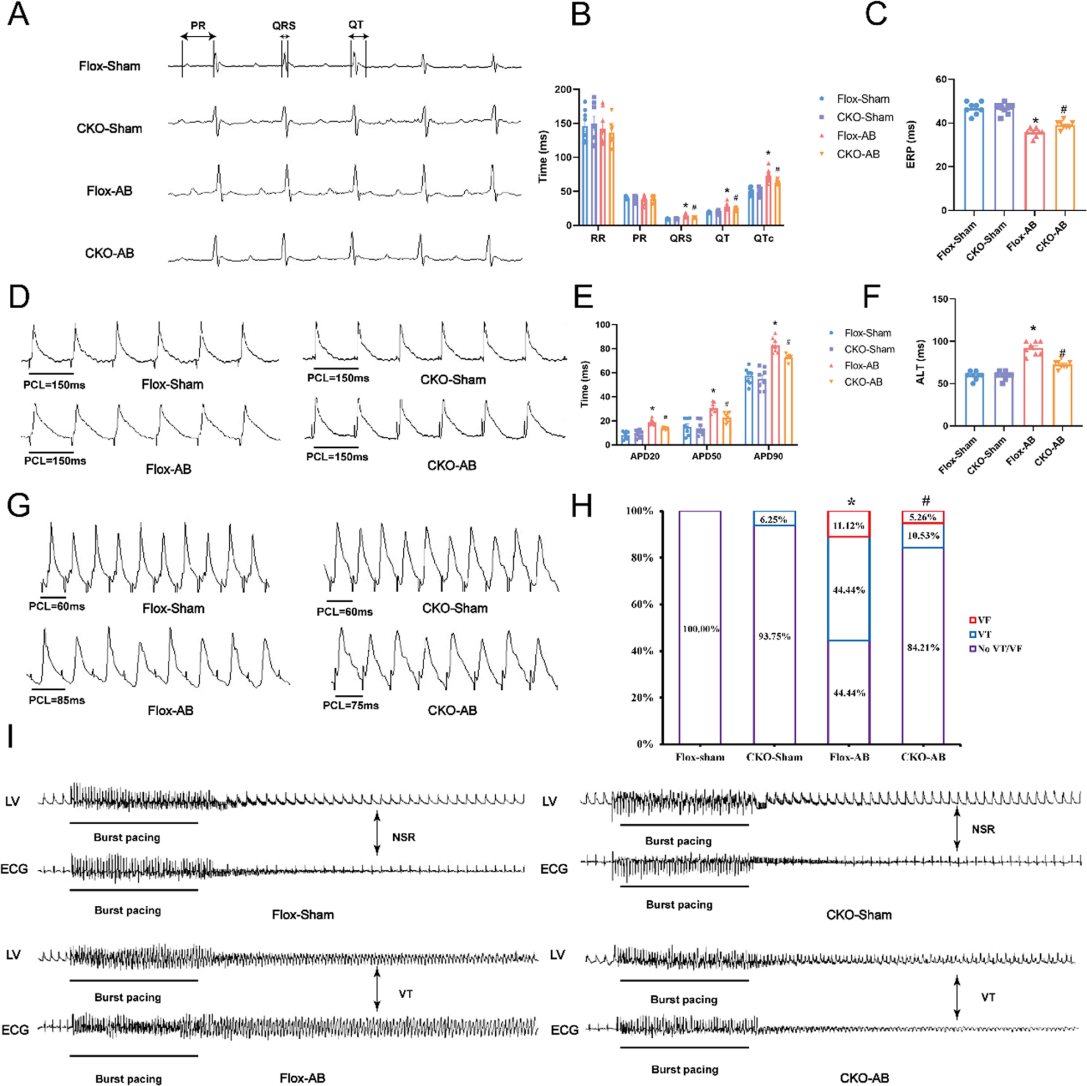
USP38 deficiency reduces the susceptibility of VAs in mice four weeks after the AB surgery. **A**, **B** Representative electrocardiograph and statistical analysis of RR, PR, QRS, QT interval, and QTc (n = 8). **C** Representative recordings of ERP (n = 8). (D-E) Representative recordings and statistical analysis of APD90, APD50 and APD20 (n = 8). **F**, **G** Representative recordings and statistical analysis of ALT (n = 8). **H**, **I** Representative ECG and left ventricle action potential changes induced by burst stimulation and statistical analysis of induction rate of VAs (n = 16–19). *P < 0.05 vs. Flox-Sham group, ^#^P < 0.05 vs. Flox-AB group

To better elucidate the role of USP38 in regulation of HF-related VAs, we utilized the Cre-LoxP system to generate USP38-CKO mice, with their wild-type littermates referred to as the Flox-Sham group. The absence of USP38 expression in the USP38-CKO mice were confirmed by Western blotting (Fig. S3A). Four weeks after AB surgery, USP38 deficiency further improved LVEF and LVFS (Fig. S3C-D). Surface ECG recordings revealed no difference in RR intervals and PR intervals between the HF with USP38 deficiency and without. After AB surgery, the Flox group exhibited a significant increase in QRS duration and QTc, However, these changes were attenuated in the USP38-CKO group following AB surgery (Fig. 2A, B).

In vivo electrophysiology was analyzed to characterize ventricular electrophysiological parameters, including ERP, APD and ALT. Compared with Flox-Sham mice, the Flox-AB group showed marked prolongation in APD20, APD50, APD90, and ALT; however, USP38 knockout reversed these changes (Fig. 2D–G). Additionally, the ERP was shorter in Flox group after AB surgery, whlie USP38 knockout suppressed the decrease in ERP (Fig. 2C). Moreover, our results revealed that the induction rate of VAs in the Flox-AB group was significantly higher compared to the Flox-Sham group, with VT accounting for 44.44% and VF for 11.12%. Conversely, CKO-AB significantly reduced the rates of VAs induction compared to the Flox-AB group, with VT accounting for 10.53% and VF for 5.26% (Fig. 2H, I). These findings suggest that USP38 deficiency reduces vulnerability to VAs in HF after AB surgery.

### USP38 overexpression aggravates cardiac function increases the susceptibility of VAs in mice with HF induced by pressure overload.

**Fig. 3:**
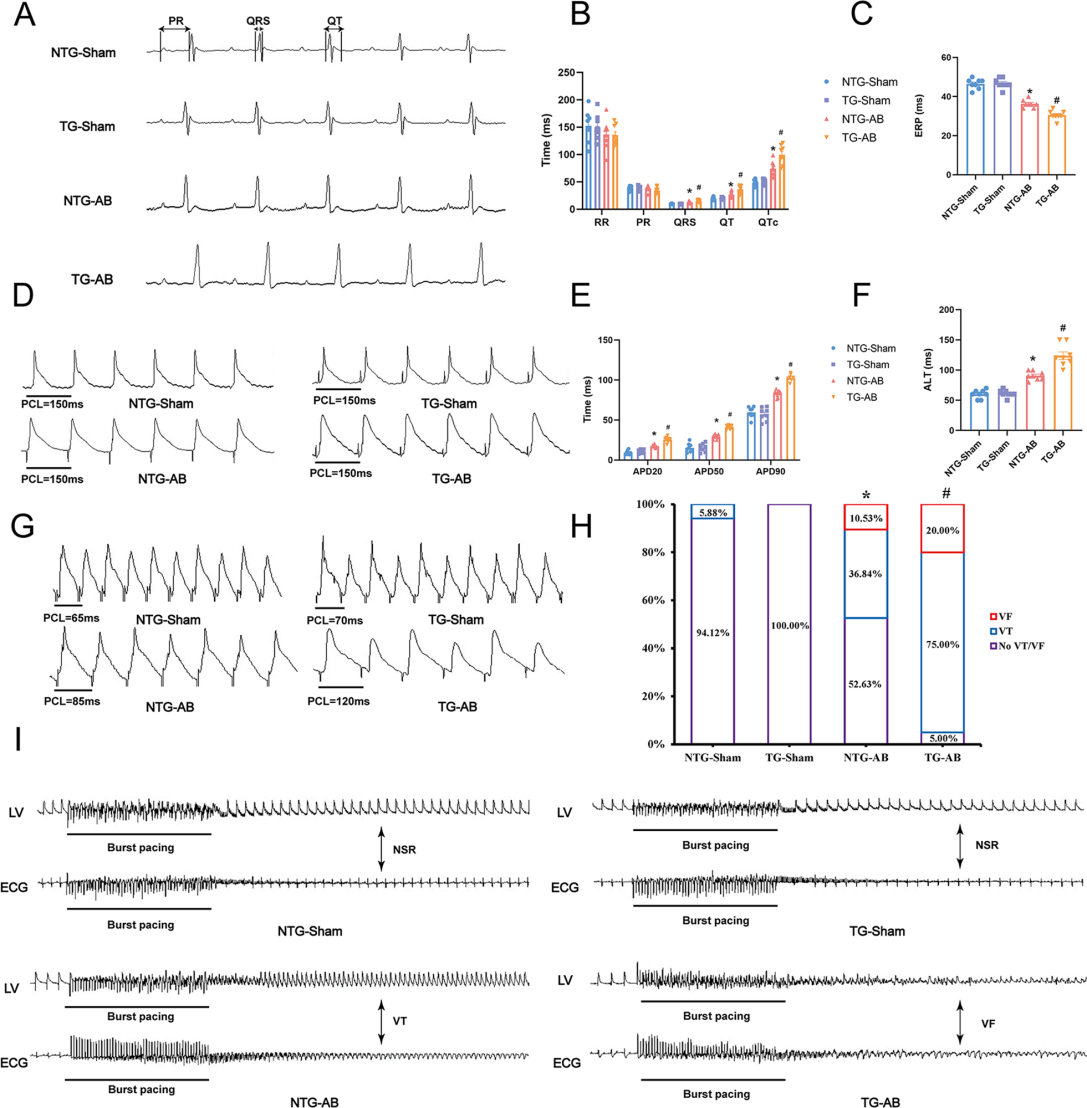
USP38 overexpression increases the susceptibility of VAs in mice four weeks after the AB surgery. **A**, **B** Representative electrocardiograph and statistical analysis of RR, PR, QRS, QT interval, and QTc (n = 8). **C** Representative recordings of ERP (n = 8). **D**, **E** Representative recordings and statistical analysis of APD90, APD50 and APD20 (n = 8). **F**, **G** Representative recordings and statistical analysis of ALT (n = 8). **H**, **I** Representative ECG and left ventricle action potential changes induced by burst stimulation and statistical analysis of induction rate of VAs (n = 16–20). *P < 0.05 vs. NTG-Sham group, ^#^P < 0.05 vs. NTG-AB group

Next, we also established cardiac-specific USP38 overexpression mice to further verify the role of USP38 in HF induced by pressure overload (Fig. S3B). USP38 overexpression further reduced LVEF and LVFS in mice undergoing AB surgery (Fig. S3E-F). Four weeks after AB surgery, the QRS duration and QTc of the USP38-TG group and NTG group were prolonged, with the prolongation in the USP38-TG group being more pronounced than that in the NTG group (Fig. 3A, B). Meanwhile, USP38 overexpression prolonged APD20, APD50, APD90, ALT, and shorten ERP (Fig. 3C–G). Additionally, we observed a significant increase in the incidence of VAs after AB surgery in the NTG group, with VT accounting for 36.84% and VF for 10.53%. However, mice overexpressed USP38 had a higher incidence of VAs, with VT accounting for 75% and VF accounting for 20% (Fig. 3H, I). Together, these findings support the notion that USP38 overexpression increases vulnerability to VAs in HF induced pressure overload.

### USP38 reduces the expression of ion channels in mice with HF induced by pressure overload.

**Fig. 4:**
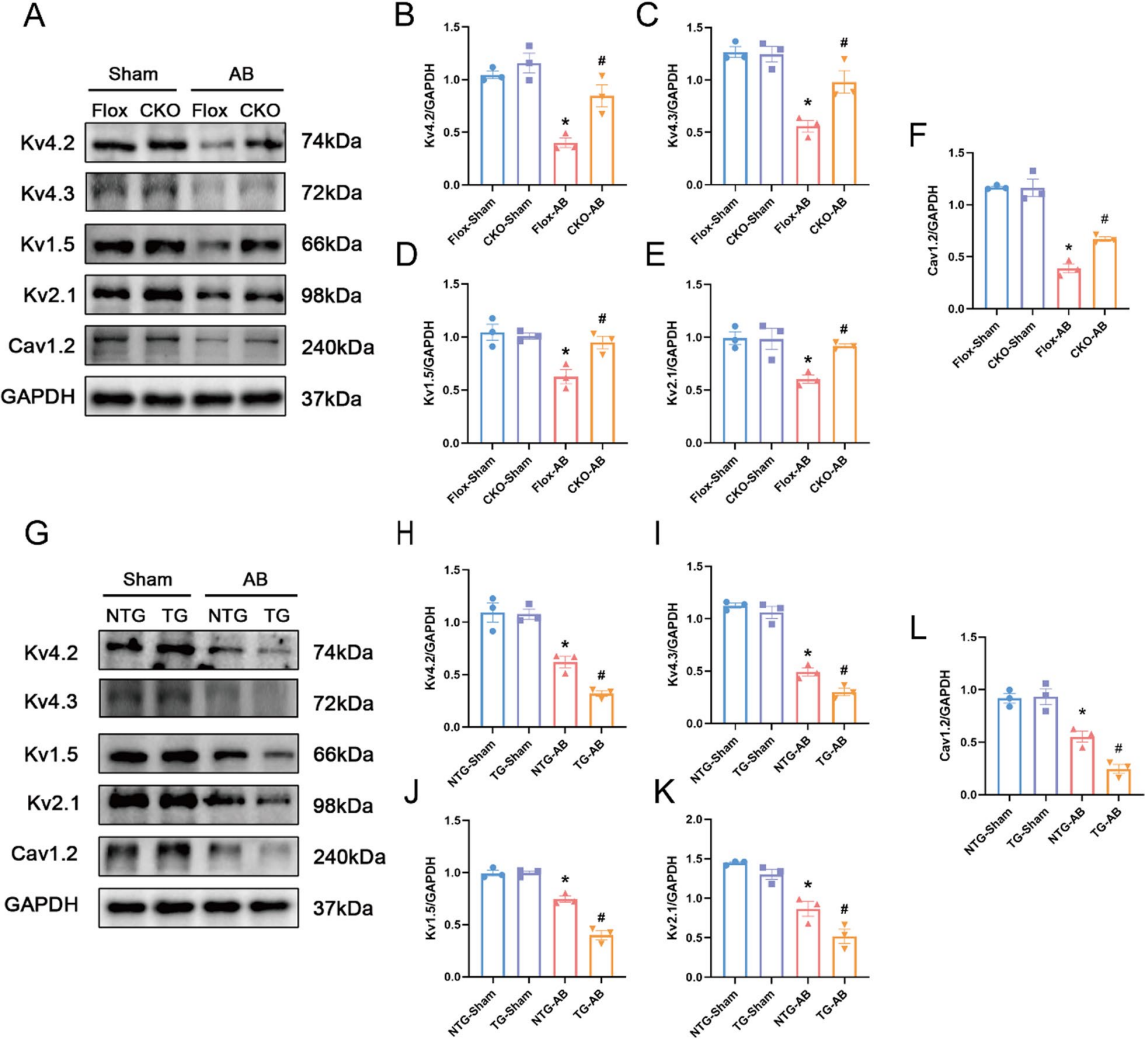
USP38 reduces the expression of ion channels in mice four weeks after the AB surgery. **A** Representative Western blots of the ion channel proteins levels (Kv4.2, Kv4.3, Kv2.1, Kv1.5, and Cav1.2) in cardiac-conditional USP38 knockout mice (n = 3). **B**–**F** Statistical analysis of the ion channel protein levels in the four groups 4 weeks after AB surgery (n = 3). *P < 0.05 vs. Flox-Sham group, ^#^P < 0.05 vs. Flox-AB group. **G** Representative Western blots of the ion channel proteins levels (Kv4.2, Kv4.3, Kv2.1, Kv1.5, and Cav1.2) in cardiac-specific USP38 overexpression mice (n = 3). **H**–**L** Statistical analysis of the ion channel protein levels in the four groups 4 weeks after AB surgery (n = 3). *P < 0.05 vs. NTG-Sham group, # P < 0.05 vs. NTG-AB group

The abnormal expression of ion channel is closely related to VAs, so we assessed the protein expression of Ito, f channels (Kv4.3, Kv4.2) and IK, slow channels (Kv2.1 and Kv1.5). The protein levels of Kv4.3 and Kv4.2 decreased significantly in HF mice after AB surgery. Additionally, the protein levels of Kv2.1 and Kv1.5 were also markedly reduced in HF mice. Following AB surgery, the protein expression of Cav1.2, the pore-forming subunit of ICa-L in cardiac tissue, was significantly decreased compared to the Flox-Sham group. The USP38-CKO group showed an improvement in the expression level of the above channel proteins (Fig. 4A–F). There results suggest that USP38 deletion could up-regulate the expression of cardiac ion channel protein.

To further explore the correlation between the prolongation of action potential and ion channels, we investigated the protein expression levels of ion channels in mice with USP38 overexpression. After 4 weeks of AB surgery, the expression of Kv4.3, Kv4.2 and Kv2.1, Kv1.5 in USP38-TG and NTG groups decreased drastically, but these changes in USP38-TG hearts were considerably greater than those in NTG hearts (Fig. 4G–K). Moreover, the protein expression of ICaL was significantly lower in USP38-TG group than in the NTG group after AB surgery (Fig. 4G, L). The above results demonstrated that USP38 overexpression could down-regulate the expression of cardiac ion channel protein after AB surgery.

### USP38 decreases the expression of Cx43 in HF induced by pressure overload.

**Fig. 5:**
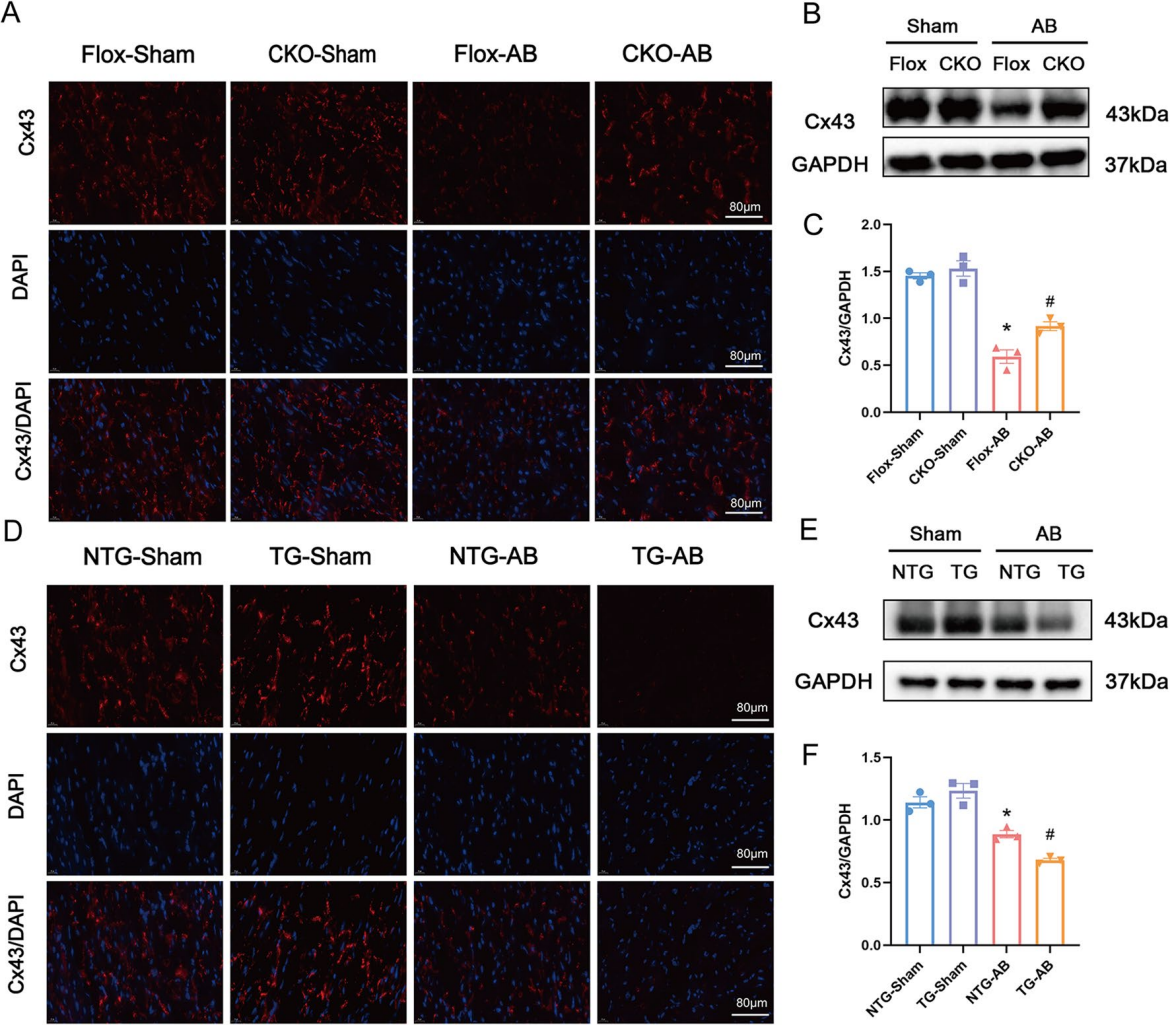
USP38 reduces the expression of Cx43 in mice four weeks after the AB surgery. **A** The left ventricle of mice was stained with anti-Cx43 antibody (red) and DAPI (blue). Representative Cx43 immunofluorescence staining images in cardiac-conditional USP38 knockout mice (n = 4). **B**, **C** Representative Western blotting and statistical analysis of Cx43 in cardiac-conditional USP38 knockout mice (n = 3). * P < 0.05 vs. Flox-Sham group, # P < 0.05 vs. Flox-AB group. **D** Representative Cx43 immunofluorescence staining images in cardiac-specific USP38 overexpression mice (n = 4). **E**, **F** Representative western blotting and statistical analysis of Cx43 in cardiac-specific USP38 overexpression mice (n = 3). *P < 0.05 vs. NTG-Sham group, # P < 0.05 vs. NTG-AB group

Cardiac remodeling is typically accompanied by changes in the expression of Cx43. To explore the role of Cx43 protein in the regulation of VAs by USP38, we determined the expression of cardiac Cx43 induced by pressure overload using immunofluorescence analysis. In the Flox-AB group, the expression of Cx43 significantly decreased, while USP38 deletion improved these changes (Fig. 5A–C). Similarly, we analyzed the expression of Cx43 in USP38 overexpression group by immunofluorescence analysis and Western blot. The expression of Cx43 was significantly decreased in NTG mice with heart failure induced by pressure overload. Further findings revealed a significant decrease in the protein level of Cx43 in ventricular tissue in USP38-TG mice after AB surgery compared to NTG-AB mice (Fig. 5D–F).

### USP38 regulates TBK1/AKT/CaMKII signaling pathway.

**Fig. 6:**
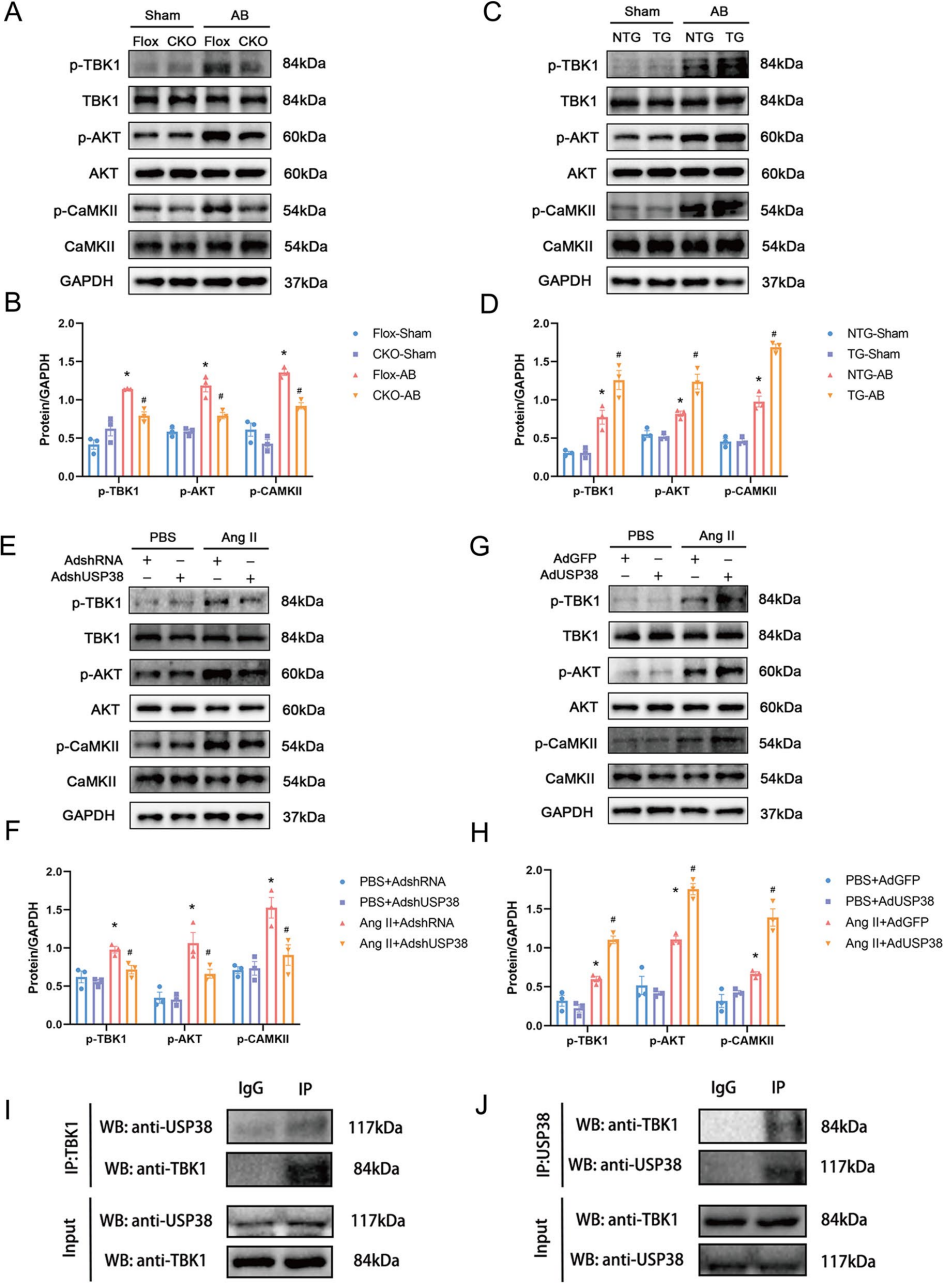
USP38 regulates TBK1/AKT/CaMKII signaling pathway. **A**, **B** Representative western blotting and statistical analysis of p-TBK1, p-AKT and p-CAMKII in cardiac-conditional USP38 knockout mice (n = 3). *P < 0.05 vs. Flox-Sham group, # P < 0.05 vs. Flox-AB group. **C**, **D** Representative western blotting and statistical analysis of p-TBK1, p-AKT and p-CAMKII in cardiac-specific USP38 overexpression mice (n = 3). *P < 0.05 vs. NTG-Sham group, # P < 0.05 vs. NTG-AB group. (**E**–**F**) Representative western blotting and statistical analysis of p-TBK1, p-AKT and p-CAMKII in H9c2 cells infected by AdshRNA and AdshUSP38 (n = 3). *P < 0.05 vs. PBS+AdshRNA group, ^#^P < 0.05 vs. AngII + AdshRNA group. (**G**-**H**) Representative western blotting and statistical analysis of p-TBK1, p-AKT and p-CAMKII in H9c2 cells infected by AdGFP and AdUSP38 (n = 3). *P < 0.05 vs. PBS+AdGFP group, ^#^P < 0.05 vs. AngII + AdGFP group. **I**, **J** Endogenous immunoprecipitation analysis of the interaction between USP38 and TBK1 in H9c2 cells using anti-IgG, anti-USP38, or anti-TBK1 (n = 3)

Increasingly evidence suggests that TBK1, AKT, and CaMKII, identified as regulators, play crucial roles in cardiac remodeling (Mustroph et al. 2017; Jiang et al. 2015). Our data indicated a significant increase in the phosphorylation of TBK1 and its downstream targets, including AKT and CaMKII, in response to pressure overload stimuli in ventricular tissue due to USP38 overexpression. Conversely, the activation of phosphorylated forms of these proteins was impeded by USP38 deficiency (Fig. 6A–D). In in vitro experiments, H9c2 cells were infected with AdshUSP38 to knockdown USP38 or with AdUSP38 to overexpress USP38, followed by treatment with 1uM Ang II. H9c2 cells infected with AdshRNA and AdGFP were used as the control group. The levels of phosphorylated TBK1, AKT, and CaMKII significantly increased in Ang II-stimulated cells. The Ang II-induced elevation in phosphorylated TBK1, AKT, and CAMKII expression was notably blocked in cells with USP38 deficiency compared to control cells, whereas USP38-overexpressing cells exhibited higher levels of phosphorylated TBK1, AKT, and CaMKII in response to Ang II stimuli (Fig. 6E–H). Finally, we examined the interaction of USP38 with TBK1 by co-immunoprecipitation (Fig. 6I, J). Together, these results demonstrate that USP38 promotes the activation of the TBK1/AKT/CaMKII signaling pathway in mice with HF.

## Discussion

In this study, we observed the following: (1) Expression of USP38 was increased in HF induced by pressure overload; (2) USP38 overexpression exacerbated susceptibility to VAs after HF, as indicated by prolonged QRS intervals and QTc, extended APD, shortened ERP, and a higher incidence of VAs; (3) USP38 deficiency reduced susceptibility to VAs after HF, prolonged ERP, and shortened QRS intervals, QTc, and APD; (4) USP38 deficiency increased, while USP38 overexpression decreased ion channels and Cx43 in HF induced by pressure overload. These findings implied that USP38 influenced cardiac electrical remodeling after HF through the TBK1/AKT/CAMKII signaling pathway, thereby increasing the Susceptibility of VAs after HF.

The ubiquitin–proteasome system (UPS) regulates protein degradation, cell signaling, and other cellular processes through a reversible process to target proteins. USP plays a regulatory role in cardiac diseases such as myocardial infarction, cardiac hypertrophy, and ischemic heart disease (Song et al. 2022; Heitmeier et al. 2020). Although the role of USP38 in tumors, respiratory diseases and myocardial infarction has been previously studied, its role in VAs after HF has not yet been completely elucidated (Gong et al. 2023). This study found that the USP38 was highly expressed in the left ventricle of HF mice, and Ang II-induced in vitro also revealed an upregulation of USP38 protein levels.

Clinical research has showed that VAs is common and a primary cause of mortality in HF patients (Desai et al. 2022). With the deterioration of heart function in HF, the risk of VAs and the associated occurrence of sudden cardiac death (SCD) markedly increase (Curtain et al. 2023). The prolongation of QRS intervals and QTc indicates abnormal cardiac electrophysiology and is associated with intraventricular conduction disorders, which increase the risk of VAs and SCD (Desai et al. 2022; Miró et al. 2023; Tse and Yan 2017). Therefore, we speculate that USP38 is related to VAs after HF. Our results confirmed that QRS and QTc were prolonged in mice after AB surgery, and USP38 overexpression aggravated these ECG parameters, but USP38 deficiency shortened the above parameters. The prolongation of APD, as a substrate for arrhythmia, can induce the formation of early afterdepolarizations and trigger arrhythmias. In previous studies, the increased vulnerability of VAs after HF was associated with the prolongation of APD and ALT, and the shortening of ERP (Peng et al. 2017). Like previous studies, the results of our study showed that the induction rate of VAs is increased in TG-AB mice, which may be related to the fact that USP38 overexpression can aggravate the prolongation of APD90 and the shortening of ERP. Deletion of USP38 alleviates these electrophysiological changes.

Electrical remodeling is a pathological mechanism in the evolution of VAs, wherein ion channel abnormalities play a crucial role. The generation of ventricular action potentials relies on myocardial ion channels. Ito, f (Kv4.2, Kv4.3) and IK, slow (Kv2.1 and Kv1.5) represent distinct subtypes of voltage-gated potassium channels that undergo opening or closing based on changes in membrane potential (Shuai et al. 2020). Downregulation of these ion channels increases the risk of VAs by prolonging ventricular action potential duration and slowing conduction velocity. In USP38 knockout heart tissue, the expression Kv4.2, Kv4.3, Kv2.1 and Kv1.5 are increased. In addition, USP38 overexpression has the opposite effect, reducing the expression of Kv4.2, Kv4.3, Kv2.1 and Kv1.5. Several studies have demonstrated reduced levels of Cav1.2 in HF (Lu et al. 2017). The L-type calcium channel (Cav1.2), generating inward current, promotes second-phase depolarization and shortens the duration of the action potential (Keefe et al. 2023). This experiment observed that USP38 overexpression downregulated the expression level of Cav1.2, and the prolongation of APD may be attributed to the reduction of outward potassium current, exerting a stronger impact on APD. These findings were further confirmed in our study, indicating that USP38 decreases the expression of ion channels in mice after HF, thereby prolonging action potential duration and increasing susceptibility to VAs.

Cx43 exists in the ventricular gap junction structure, regulating electrical conduction between cardiomyocytes and coordinating heart rhythm. Some studies have confirmed that the decreased expression and disordered distribution of Cx43 in HF are important driving factors for VAs (Zheng et al. 2023; Wu et al. 2022). In addition, Cx43 can also regulate calcium ions, contributing to arrhythmia (Boulaksil et al. 2010). Our study revealed that the expression level and distribution area of Cx43 in the ventricle were further decreased after USP38 overexpression in mice with HF, while USP38 knockout inhibited the degradation of Cx43. Therefore, our findings suggest that USP38 may increase susceptibility to VAs occurring after HF by downregulating Cx43 expression in the ventricular myocardium.

Huang et al. previously demonstrated that USP38 regulates TBK1 to maintain immune homeostasis (Lin et al. 2016). The negative regulator of the interferon pathway can inhibit the TBK1/AKT signaling pathway, reducing cardiac remodeling and the risk of HF (Deng et al. 2016). In addition, activation of AKT can lead to dysfunction in calcium regulatory proteins and the activation of CaMKII, closely linked to the occurrence of arrhythmias (Curran et al. 2014). Consistent with previous findings, this study revealed a significant increase in TBK1, AKT and CaMKII phosphorylation levels after pressure overload-induced HF, and these adverse effects were mitigated in USP38 knockout mice. co-immunoprecipitation suggests an interaction between USP38 and TBK1. Based on previous and our present studies, we deduce that USP38 acts on the TBK1/AKT/CaMKII signaling pathway to improve ventricular electrical remodeling after HF.

## Limitations

Undoubtedly, certain questions still require careful consideration. Primarily, the intricate landscape of VAs encompasses four principal pathophysiological mechanisms: electrical remodeling, structural remodeling, autonomic nervous system changes, and Ca2 + -handling abnormalities. Notably, our current investigation delves exclusively into the realm of electrical remodeling induced by HF. Further exploration is imperative to ascertain whether USP38 regulates additional mechanisms. This intricate link remains an avenue for exploration in forthcoming studies. Finally, it is crucial to acknowledge the inherent challenges associated with extrapolating data derived from murine models to human physiology. To fortify our assertions regarding the translational application of USP38, future studies should incorporate clinical research experiments.

## Conclusion

Based on these data, we conclude that USP38 increases vulnerability to VAs after HF through the TBK1/AKT/CaMKII signaling pathway. USP38 may be an attractive therapeutic target for VAs associated with pressure overload-induced HF.

### Supplementary Information

Supplementary Material 1.

## Data Availability

Data relevant to this study are available upon reasonable request from the corresponding author.
