# Supplementary material for: USP38 exacerbates pressure overload-induced left ventricular electrical remodeling
Source: Mol Med. 2024 Jun 27;30:97. doi: 10.1186/s10020-024-00846-3 (PMC11210128; doi:10.1186/s10020-024-00846-3)
Supplement: Supplementary file 1 — Supplementary Material 1. [file 10020_2024_846_MOESM1_ESM.docx]

***Supplemental Material***

**USP38 exacerbates pressure overload-induced left ventricular electrical remodeling.**

**Supplementary Table 1**

**Table S1 Sequence of primers used for quantitative real-time polymerase chain reaction (qRT-PCR).**

| Genes | Species | Sequence (5’ → 3’) |
| --- | --- | --- |
| USP38 | Rat | (Forward)-AAGGAGCTCCGGGAGTATGT  (Reverse)-GAAGGCAGGCCATCACTTCT |
| USP38 | Mouse | (Forward)- TCAATCAAAGCGCCTGGACT  (Reverse)- CCCACAGTTTAGGCAGCAGA |
| GAPDH | Rat | (Forward)- GAAGGTCGGTGTGAACGGAT  (Reverse)- CCCATTTGATGTTAGCGGGAT |
| GAPDH | Mouse | (Forward)-ACTCCACTCACGGCAAATTC  (Reverse)-TCTCCATGGTGGTGAAGACA |

**Supplementary Fig. 1**


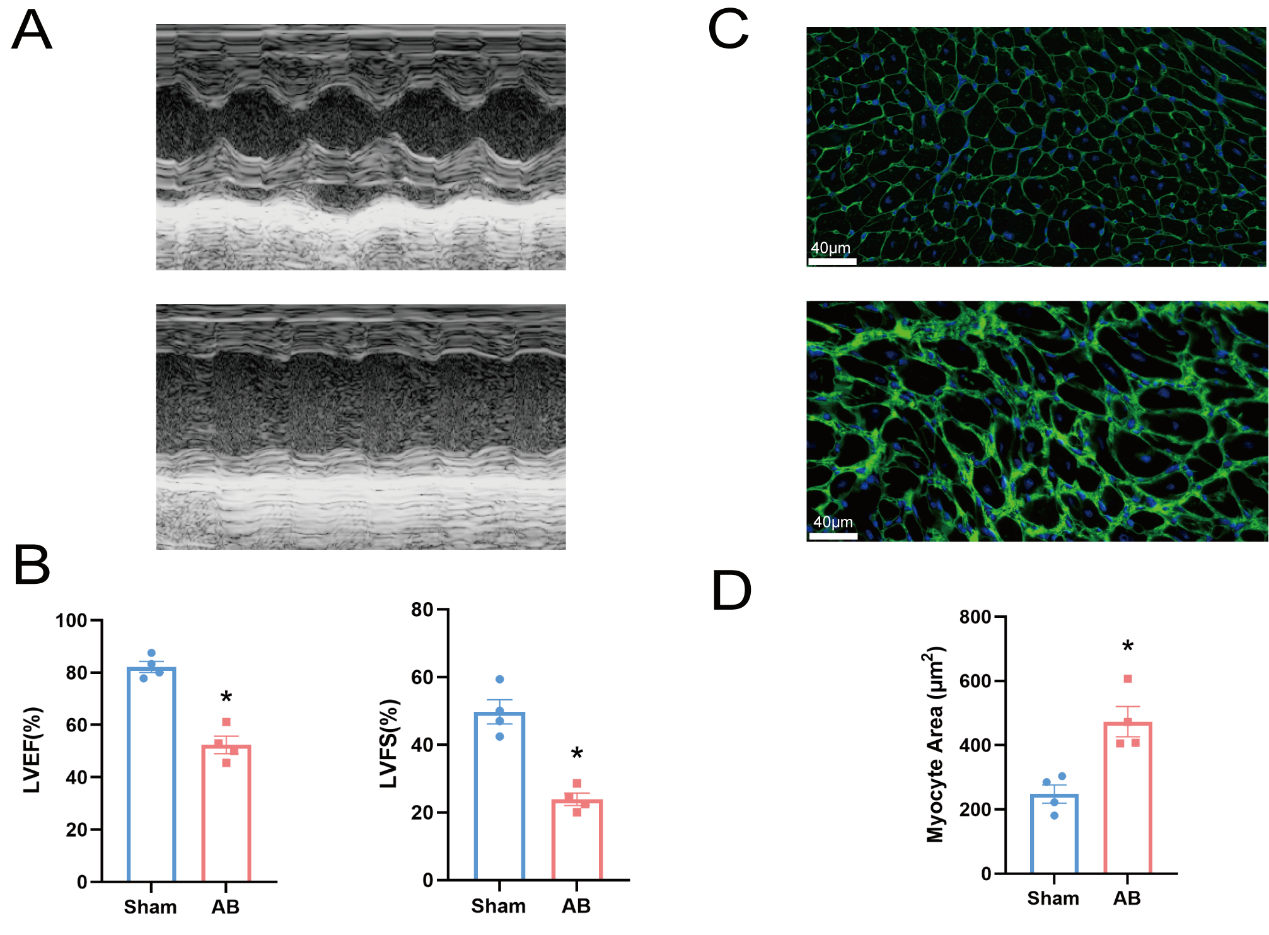


**Fig. S1. Changes of cardiac function and morphology in mice four weeks after AB surgery.** (A) Representative echocardiographic images of mice at four weeks after AB surgery. (B) statistical analysis of LVEF and LVFS (n=4). (C) Representative WGA staining of the mice at four weeks after AB surgery (D) statistical analysis of WGA staining (n=4). * P < 0.05 vs. Sham group.

**Supplementary Fig. 2**


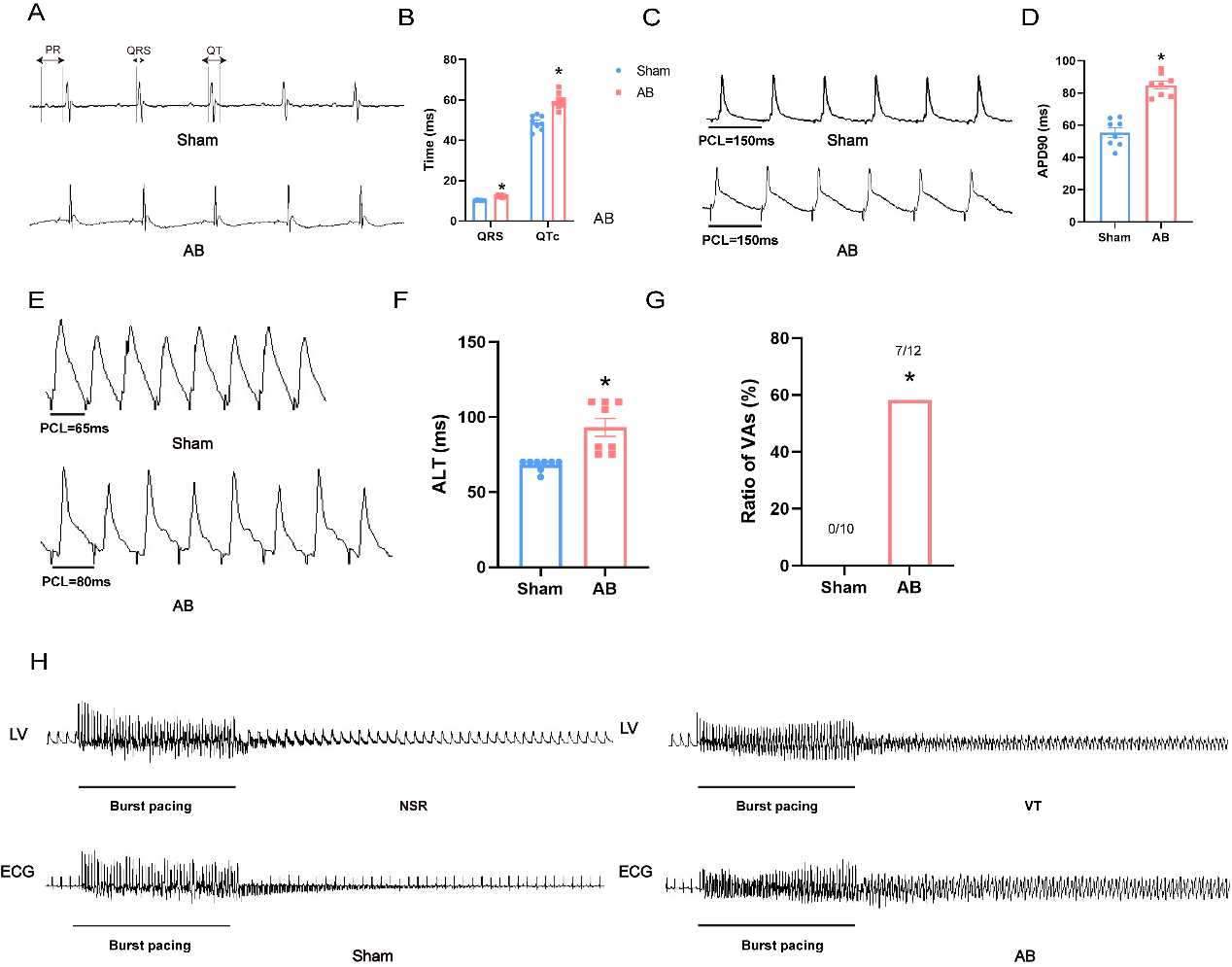


**Fig. S2. Increased susceptibility to VAs in mice four weeks after AB.** (A-B) Representative recordings and statistical analysis of ECG parameters (n=8). (C-D) Representative recordings and statistical analysis of APD (n=8). (E-F) Representative recordings and statistical analysis of ALT (n=8). (G-H) Representative ECG and left ventricle action potential changes induced by burst stimulation and statistical analysis of induction rate of VAs (n=10-12). * P < 0.05 vs. Sham group.

**Supplementary Fig. 3**

**
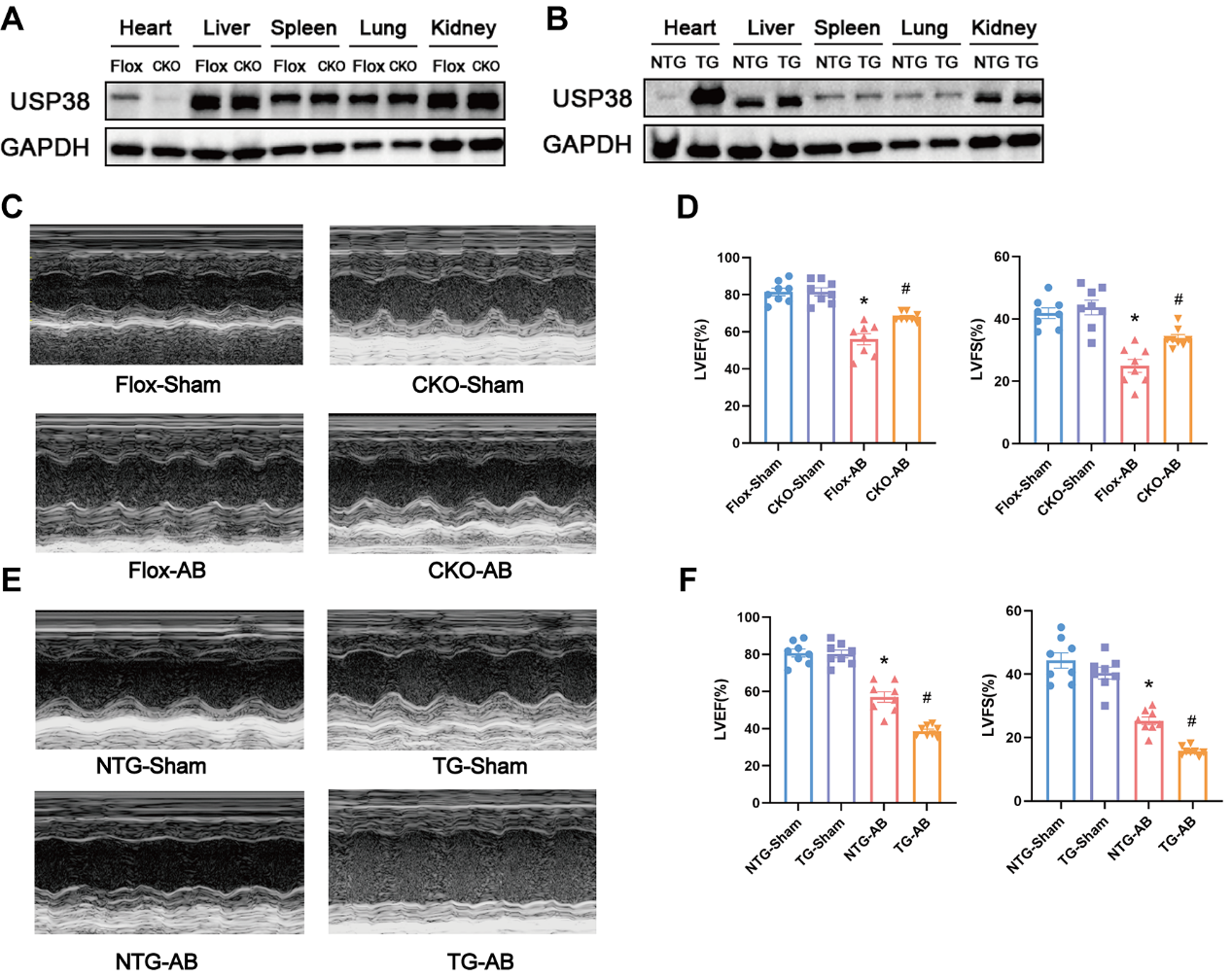
**

**Fig. S3. USP38 aggravated pressure overload induced ventricular function.** (A) Representative immunoblotting of USP38 protein in the left ventricle tissues from Flox and CKO mice (n=3). (B) Representative immunoblotting of USP38 protein in the left ventricle tissues from NTG and TG mice (n=3). (C-D) Representative echocardiographic images and statistical analysis of left ventricular ejection fraction and left ventricular fraction shortening of Flox and CKO mice at 4 weeks after sham or AB surgery (n=8). * P<0.05 vs. Flox-Sham group, # P<0.05 vs. Flox-AB group. (E-F) Representative echocardiographic images and statistical analysis of left ventricular ejection fraction and left ventricular fraction shortening of NTG and TG mice at 4 weeks after sham or AB surgery (n=8). * P<0.05 vs. NTG-Sham group, # P<0.05 vs. NTG-AB group.
